# Mapping discontinuous epitopes for MRK-16, UIC2 and 4E3 antibodies to extracellular loops 1 and 4 of human P-glycoprotein

**DOI:** 10.1038/s41598-018-30984-8

**Published:** 2018-08-24

**Authors:** Shahrooz Vahedi, Sabrina Lusvarghi, Kristen Pluchino, Yinon Shafrir, Stewart R. Durell, Michael M. Gottesman, Suresh V. Ambudkar

**Affiliations:** Laboratory of Cell Biology, Center for Cancer Research, National Cancer Institute, National Institutes of Health, Bethesda, Maryland 20892-4256 USA

## Abstract

P-glycoprotein (P-gp), an ATP-dependent efflux pump, is associated with the development of multidrug resistance in cancer cells. Antibody-mediated blockade of human P-gp activity has been shown to overcome drug resistance by re-sensitizing resistant cancer cells to anticancer drugs. Despite the potential clinical application of this finding, the epitopes of the three human P-gp-specific monoclonal antibodies MRK-16, UIC2 and 4E3, which bind to the extracellular loops (ECLs) have not yet been mapped. By generating human-mouse P-gp chimeras, we mapped the epitopes of these antibodies to ECLs 1 and 4. We then identified key amino acids in these regions by replacing mouse residues with homologous human P-gp residues to recover binding of antibodies to the mouse P-gp. We found that changing a total of ten residues, five each in ECL1 and ECL4, was sufficient to recover binding of both MRK-16 and 4E3 antibodies, suggesting a common epitope. However, recovery of the conformation-sensitive UIC2 epitope required replacement of thirteen residues in ECL1 and the same five residues replaced in the ECL4 for MRK-16 and 4E3 binding. These results demonstrate that discontinuous epitopes for MRK-16, UIC2 and 4E3 are located in the same regions of ECL1 and 4 of the multidrug transporter.

## Introduction

Of 48 known human ABC (ATP-binding cassette) transporters, ABCB1, also known as P-glycoprotein (P-gp) or multidrug resistance 1 (MDR1), is the most studied protein of this superfamily^1,2^. P-gp is an ATP-dependent transporter that protects cells by effluxing a broad range of xenobiotic substances out of cells^1^. P-gp is expressed on the lumenal surface of epithelial cells lining the intestine, kidney, and liver and pumps out harmful agents into the feces, urine, and bile, respectively^3,4^. It has been shown clinically that tumors that express a high level of P-gp respond poorly to chemotherapy^5,6^. An increase in P-gp expression in cancer cells, due to frequent genetic abnormalities and/or exposure to chemotherapeutic drugs, has been linked to the development of multidrug resistance^1–7^.

**Figure 1:**
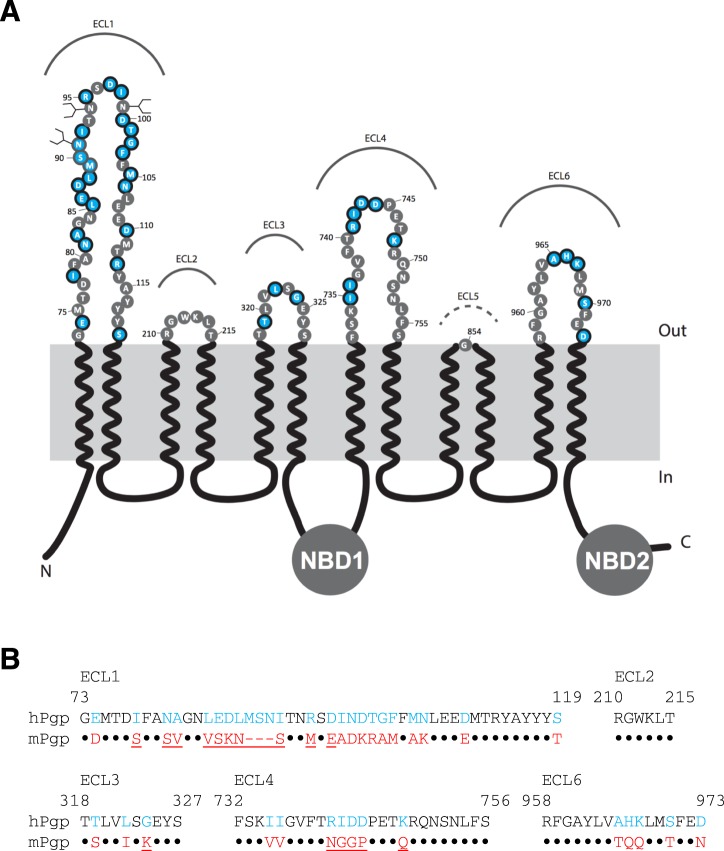
Topology and sequence comparison of the extracellular loops of human and mouse P-gp. (**A**) Schematic representation of the primary structure of human P-gp. ECLs 1 to 6 are labeled. Residues that are different in human and mouse P-gps are highlighted in light blue. (**B**) Sequence alignment of putative human and mouse P-gp extracellular loop residues. The ECL5 was omitted due to its shortness and sequence conservation. Residues M89, S90 and N91 are not present in mouse P-gp. The residues that are different in human and mouse P-gp are shown in blue and red, respectively. Conserved residues in both human mouse transporters are presented as black dots. The mutated residues in the ECL regions of mouse P-gp are underlined.

Structurally, like many ABC transporters, P-gp has twelve transmembrane helices (TMHs) organized in two transmembrane domains, which recognize various structurally and chemically unrelated drugs^8–12^. Additionally, there are two intracellular nucleotide-binding domains (NBDs) that bind and hydrolyze ATP. The TMHs are connected in the extracellular region by six extracellular loops (ECLs) (Fig. 1A). The sequences of these loops are partially conserved between human and mouse (*mdr1a*-encoded) P-gps (Fig. 1B). In the absence of substrate and nucleotide, P-gp exists in the apo conformation in an inverted V-shape in which the NBDs are separated from each other. The binding of nucleotide and substrate imposes a series of conformational changes resulting in a V-shaped closed conformation with dimerization of NBDs and movement of TMHs and ECLs to facilitate substrate transport^6,11,12^.

One of the most efficient cancer therapies that has recently become available to cancer patients is immunotherapy^13^. A remarkable example of this type of therapy is the inhibitory effect of antibodies on Her2^14^ and immune checkpoint receptors^15^, which has been demonstrated to re-activate the immune system to attack cancer cells. While most of the effort in the field of cancer immunotherapy has been focused on immune checkpoint receptors, inhibiting the function of P-gp by antibodies could re-sensitize cancer cells to potent chemotherapy drugs which otherwise are pumped out of the cells. Indeed, *in vitro* studies show that P-gp activity can be inhibited by conformation-specific antibodies with epitopes in the extracellular loops^16–18^. There are at least three widely-used monoclonal antibodies (UIC2^17^, MRK-16^16^ and 4E3^19^) that can be used to inhibit human P-gp function (reviewed in Okochi *et al*.^20^). These antibodies are mouse IgG2a isotypes. The 4E3 antibody was developed by immunizing SJL mice with SW-1573/500 cells, which are P-gp-expressing multidrug-resistant human squamous lung carcinoma cells^19^. The MRK-16 antibody was generated by immunizing mice against adriamycin-resistant human myelogenous leukemia K-562 cells^16^, whereas UIC2 was developed by immunizations of BALB/c mice with human P-gp expressing BALB/c 3T3-1000 cells^17^. *In vitro* studies show that both MRK-16 and UIC2 antibodies can inhibit P-gp function. MRK-16 partially inhibited the transport of both vinblastine and actinomycin D in K562 multidrug-resistant cells^16^. Similarly, UIC2 binding to mouse-human chimeric P-gp has been shown to re-sensitize HeLa cells to paclitaxel at nanomolar concentrations^21^.

Despite the possible clinical use of antibodies to inhibit P-gp’s function, very little is known about the precise epitopes of these antibodies. Early attempts to identify the epitope of the MRK-16 antibody was not conclusive. By predicting the amino acid sequence of the extracellular loops and using a series of overlapping synthetic peptides, Georges *et al*. reported the importance of ECLs 1 and 4 for MRK-16 binding^22^. Removal of a segment of ECL1 (78–97) also was reported to completely abolish UIC2, but not MRK-16 binding^23,24^. Using cryo-EM, the binding of the Fab of UIC2 to mouse P-gp containing all six ECLs of the human transporter has recently been reported^21^.

In this study, by generating a series of human-mouse P-gp chimeras, we identified the antibody binding epitopes for MRK-16, UIC2 and 4E3. By replacing specific mouse amino acids in ECL1 and ECL4 with human P-gp residues, we determined the amino acids required for the binding of the three antibodies. By substituting just thirteen residues in ECL1 and five in ECL4, we demonstrate the recovery of conformation-sensitive^25^ binding of UIC2 to mouse P-gp. We performed *in silico* docking of the antigen-binding fragment (Fab) region of MRK-16 and UIC2 to a homology model of human P-gp, which also supported the experimental results. Thus, we demonstrate that the discontinuous epitopes for three human P-gp-specific antibodies are overlapping and located in ECLs 1 and 4 of human P-gp.

## Results

### MRK16, UIC2 and 4E3 antibodies have conformational epitopes that are formed by extracellular loops in both halves of human P-gp

**Figure 2:**
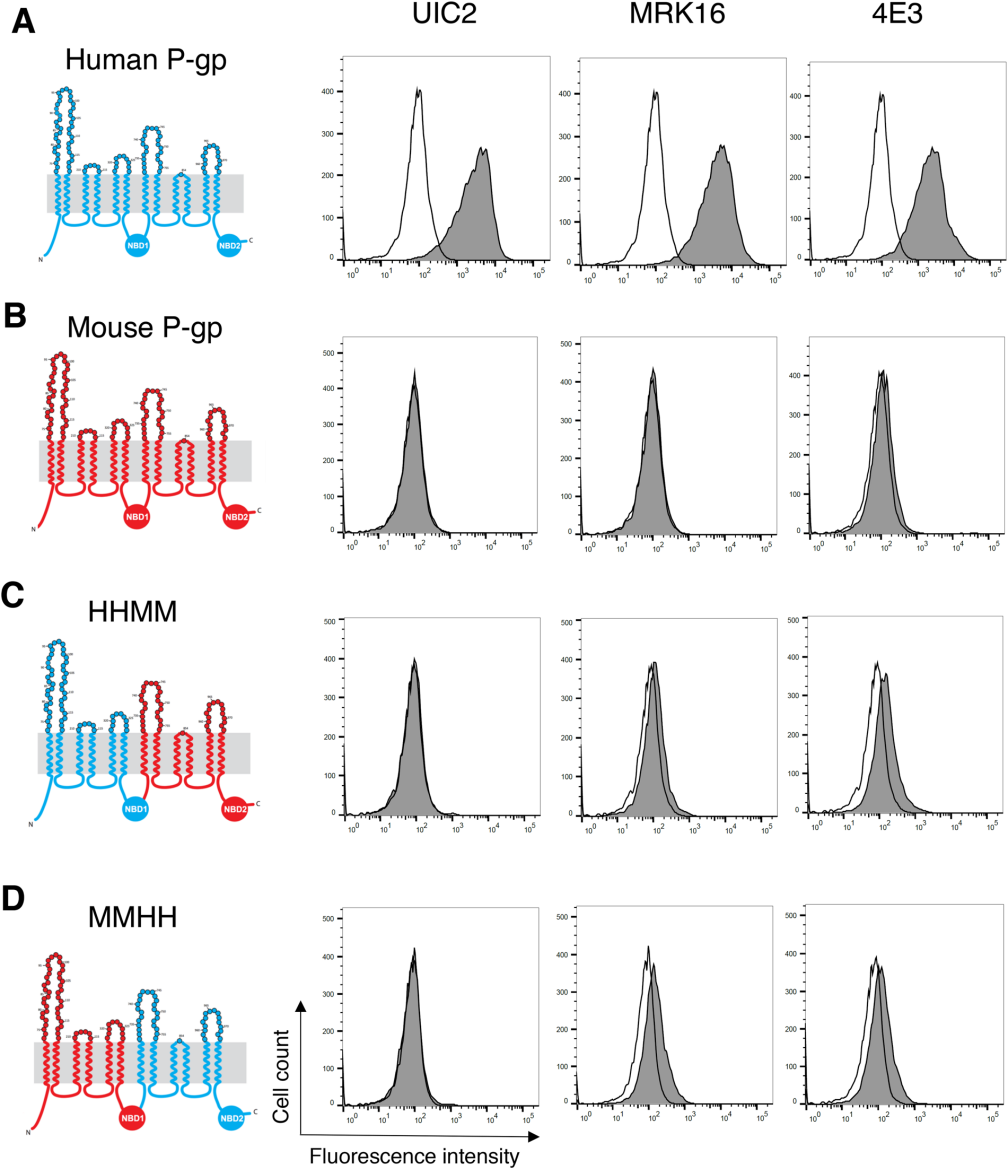
Extracellular loops from both halves of human P-gp are required for binding of the three antibodies. Schematic of the 2-D topology of P-gp (left) and representative histograms of flow cytometry analysis of antibody binding to WT human, mouse and different human-mouse P-gp chimeras are shown (right). WT human P-gp (**A**), WT mouse P-gp (**B**), HHMM (**C**), and MMHH (**D**) chimeras. The amino acids in the extracellular loops are color coded. Human-specific and mouse-specific amino acids are shown in blue and red, respectively. HeLa cells transduced with BacMam baculovirus carrying WT human, WT mouse, HHMM or MMHH P-gp chimeras were harvested 24 hours post-transduction and incubated at 37 °C with human P-gp specific antibodies (at indicated concentration per 100,000 cells) UIC2 (2 µg), MRK-16 (1 µg), and 4E3 (1.5 µg) (filled gray traces) or IgG2a control isotype (2 µg) (unfilled traces). Following incubation with primary antibodies, the cells were washed and incubated with FITC-conjugated secondary antibody at 37 °C for 30 min and the antibody binding was measured by flow cytometry (compare filled grey and unfilled traces in histograms). Similar results were obtained in three or more independent experiments.

MRK-16, UIC2 and 4E3 are three monoclonal antibodies with extracellular epitopes that specifically recognize human P-gp^16,17,23^. To verify the specificity of these antibodies, we expressed human and mouse P-gp in HeLa cells using the BacMam baculovirus system^26,27^ and tested their reactivity with the antibodies. Our data corroborated that all three antibodies reacted with human, but not mouse P-gp (Fig. 2A,B). There are four domains in P-gp structure, transmembrane domain 1 (TMD1), nucleotide-binding domain 1 (NBD1), TMD2 and NBD2. As both human and mouse P-gp homologs arise from the folding of a single polypeptide chain that is transcribed and translated in the order (N-term) TMD1-NBD1-TMD2-NBD2 (C-term), we used a four-letter code to refer to chimeras throughout the paper, where “H” corresponds to a domain based on the human amino acid sequence and “M” to a domain based on the mouse amino acid sequence. To determine whether the epitopes of any of the tested antibodies were located in the carboxy-terminal half of the molecule, we generated a chimeric P-gp in which the first half of the molecule was human (TMD1 and NBD1, HH) and the second half (TMD2 and NBD2, MM) was from mouse P-gp (HHMM chimera, Fig. 2C). We also generated an MMHH chimera, in which the first half was from mouse P-gp and the second half of the molecule was human, to test whether any of the antibody epitopes are located in the N-terminal half of the transporter (Fig. 2D). Clearly, both HHMM and MMHH chimeras were not recognized by the three antibodies. To verify if the lack of antibody signal (grey filled traces) is due to the absence of an antibody binding epitope, or altered cell surface localization of chimeric proteins, we measured the transport efficiency of human, mouse and chimeric P-gps generated in this study (Table 1) with the fluorescent substrate rhodamine 123 (Supplementary Fig. 1)^28^. Unlike untransduced HeLa cells, which show high intracellular fluorescence intensity due to the accumulation of rhodamine 123, all human-mouse P-gp chimeras efficiently effluxed rhodamine 123 out of the cells to the same level as WT human or mouse P-gp. The lack of binding of the three antibodies to MMHH and HHMM chimeric P-gps demonstrates that at least some of the residues of extracellular loops from both N- and C-terminal halves of P-gp are required for recognition by these antibodies.

**Table 1:** Reactivity of human and mouse P-gp and chimeras with human P-gp-specific MRK-16, UIC2 and 4E3 antibodies.

P-gp/Chimera	Description	Antibody binding		
		MRK-16	4E3	UIC2
Human P-gp	Human P-gp	++++	++++	++++
Mouse P-gp	Mouse P-gp	−−	−−	−−
MMHH	Mouse 1-644 (h 1-648) – human 649-1280	−−	−−	−−
HHMM	Human 1-648 – mouse 644-1270 (h 648-1280)	−−	−−	−−
HHMM-ECL4&6-h17*	TMH7/ECL4: S725(h729)A, V726(h730)I, V731(h735)I, V732(h736)I, N737(h741)R, G738(h742)I, G739(h743)D, P740(h744)D, Q7429(h748)R, I757(h761)A TMH11/ECL6/TMH12: A951(h955)G, T961(h965)A, Q962(h966)H, Q963(h967)K, T966(h970)S, N969(h973)D, I977(h981)V	++++	++++	++++
HHMM-ECL4&6-h9	ECL4: N737(h741)R, G738(h742)I, G739(h743)D, P740(h744)D, Q744(h748)K; ECL6: T961(h965)A, Q962(h966)H, Q963(h967)K, T966(h970)S	++++	+++	++++
HHMM-ECL4-h5	ECL4: N737(h741)R, G738(h742)I, G739(h743)D, P740(h744)D, Q744(h748)K	++++	++++	++++
HHMM-ECL6-h4	ECL6: T961(h965)A, Q962(h966)H, Q963(h967)K, T966(h970)S	−−	−−	−−
MMHH-ECL1-h5^#^	ECL1: S85(h86)E, 88(h89)M, 89(h89)S, 90(h90)N, M94(h95)R	+++	+++	−−
Mouse P-gp-ECL1&4-h18^#^	ECL1: S77(h78)I, S80(81)N, V81(h82)A, V84(h85)L, S85(h86)E, K86(h87)D, N87(h88)L, 88(h89)M, 89(h90)S, 90(h91)N, S91(h92)I, M94(h95)R, E96(h97)D; ECL4: N740(h741)R, G741(h742)I, G742(h743)D, P743(h744)D, Q748(h748)K	++++	++++	++++
Mouse P-gp-ECL13&4-h19^#^	ECL1: S77(h78)I, S80(81)N, V81(h82)A, V84(h85)L, S85(h86)E, K86(h87)D, N87(h88)L, 88(h89)M, 89(h90)S, 90(h91)N, S91(h92)I, M94(h95)R, E96(h97)D; ECL3: K323(h324)G; ECL4: N740(h741)R, G741(h742)I, G742(h743)D, P743(h744)D, Q748(h748)K	+++++	++++	+++++

*Contains mutations that go inside transmembrane helices 7, 11, and 12.

^#^Numbering in the final sequence is shifted by −1 compared to the human due to deletion in the mouse P-gp at position 12.

**Figure 3:**
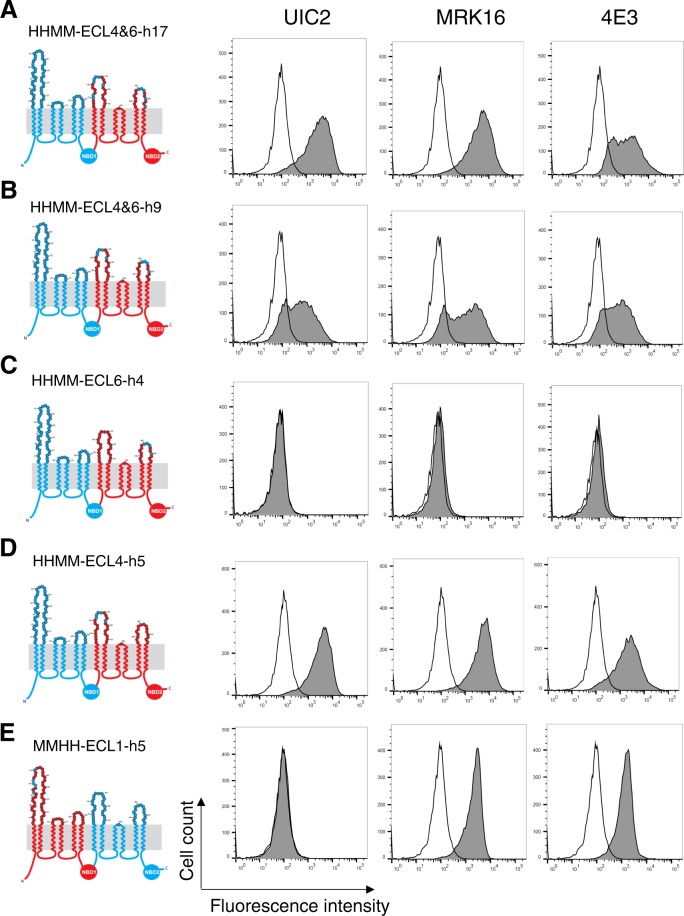
From the C-terminal half of human P-gp only, the ECL4 is required for the binding of UIC2, MRK-16 and 4E3 antibodies. The 2-D topologies of the human-mouse P-gp chimeras are depicted (left). The N-terminal halves of the first four chimeras (**A**–**D**), which include the first three extracellular loops, are human (HH) and are shown in blue. The MMHH-ECL1-h5 chimera (**E**), contains in addition to five human amino acids in ECL1 in the first half (MM), the second half of the human P-gp (HH). The number of mutations and their locations are listed in Table 1. Representative histograms of flow cytometry analysis of antibody binding to different P-gp chimeras are shown. Other details are the same as given in the legend to Fig. 2.

**Figure 4:**
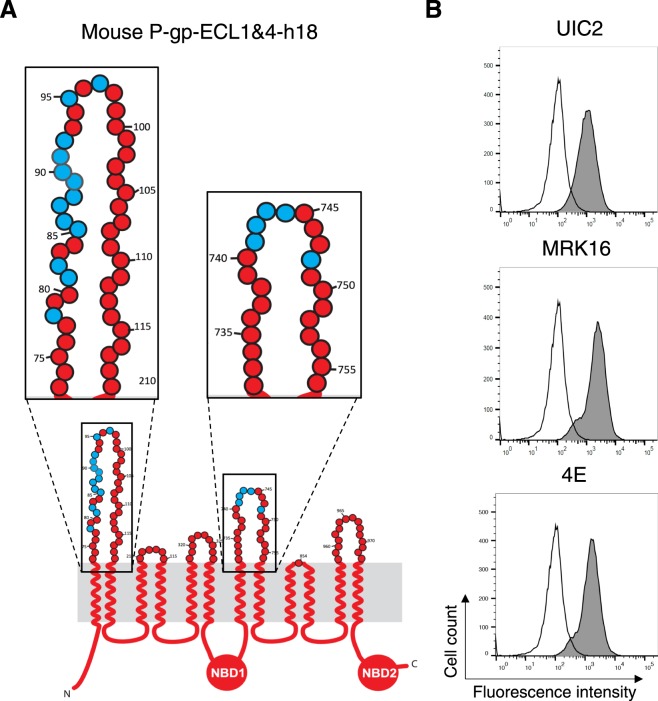
Thirteen amino acids from ECL1 and five residues from ECL4 of human P-gp are required for binding of UIC2 antibody to mouse P-gp. (**A**) Schematic of the mouse-ECL1&4-h18 chimera structure. The ECLs 1 and 4 are enlarged in the insets. Mouse-specific amino acids are in red and replaced human P-gp amino acids are shown in blue. (**B**) Representative histograms of antibody binding to the mouse-ECL 1&4-h18 chimera. Replacing thirteen residues in ECL1 and five amino acids in ECL4 of mouse P-gp completely recovered binding of the human P-gp-specific monoclonal UIC2 antibody as well as MRK-16 and 4E3.

**Figure 5:**
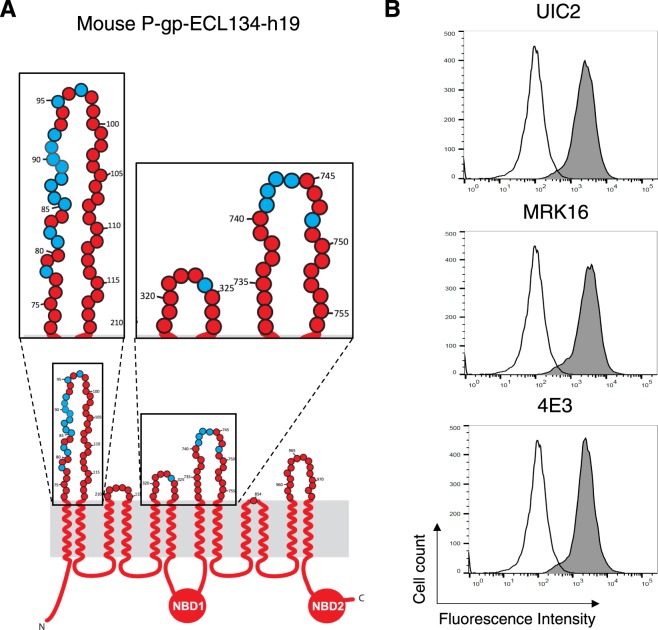
Role of K323G substitution in ECL3 of the mouse P-gp-ECL13&4-h19 chimera. (**A**) Schematic of the 2-D structure of mouse-ECL13&4-h19 chimera. The ECLs 1, 3 and 4 are enlarged in insets. (Other details are the same as given in the legend to Fig. 4). (**B**) Histograms from a representative experiment depict UIC2, MRK-16 and 4E3 antibody staining of the mouse-ECL134-h19 chimera.

**Figure 6:**
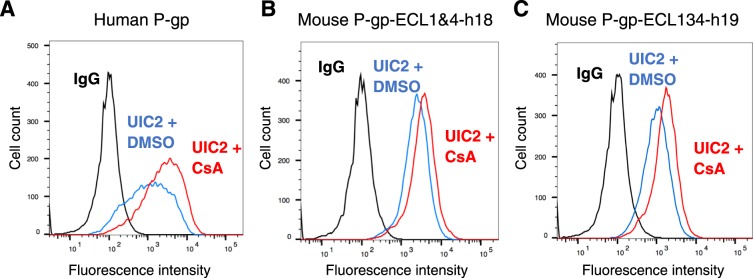
Substrate-dependent conformational changes in UIC2 binding to mouse-human P-gp chimeras are the same as in human P-gp. (**A**) WT human P-gp, (**B**) mouse P-gp-ECL1&4-h18 and (**C**) mouse P-gp-ECL13&4-h19 chimera-expressing HeLa cells were incubated for 5 minutes at 37 °C with DMSO (solvent control) or 20 µM cyclosporine A before adding UIC2 antibody (at saturated concentration, 3 µg/100,000 cells). After 30 min incubation, the cells were washed and incubated with FITC-labeled anti-mouse secondary antibody for another 30 min before acquiring data with flow cytometry. Histograms from a typical experiment are shown. The experimental details are given in each histogram. The blue traces show the UIC2 binding in the presence of DMSO and red traces depict antibody binding to cells pretreated with cyclosporine A (UIC2 shift assay). Black traces correspond to binding to the IgG control. Similar results were obtained with two additional independent experiments.

The antibody binding to each P-gp chimera was compared to human P-gp and categorized into six levels: –, no binding; +, 25%; ++, 50%, +++, 75%; ++++, 100% and +++++, >100% binding compared to human P-gp. The results are compiled from three to five independent experiments and data from a representative experiment are given in Figs 2–6. For human-mouse P-gp chimeras, the human amino acids and their numbers are given in parentheses.

### Substitution of only five mouse ECL4 residues with human residues in a human-mouse HHMM P-gp chimera is sufficient for binding of the three antibodies

After establishing that the epitopes for all three antibodies require both halves of human P-gp, we utilized a systematic approach to identify which ECLs were involved in the antibody binding and then which specific amino acids in the ECLs are critical for binding. First, we focused on identifying the amino acids forming part of the epitopes in the second half of P-gp. For this purpose, we generated four chimeras with an HHMM backbone (Fig. 3A–D). As the amino acids in and around the short ECL5 are conserved between mouse and human P-gp, we focused only on ECL4 and ECL6. Considering that these three antibodies recognize only human P-gp, we identified amino acids that are not conserved between human and mouse P-gp (Fig. 1B) and replaced ten mouse amino acids from ECL4 and the portions of the TMH7 and TMH8 with corresponding human amino acids. Additionally, in ECL6, we replaced a total of seven amino acids in the TMH11-ECL6-TMH12 region of mouse P-gp. This P-gp chimera, named HHMM-ECL4&6-h17, includes a total of seventeen human residues in the ECL4 and ECL6 of mouse P-gp. Flow cytometry analysis of stained HeLa cells expressing the HHMM-ECL4&6-h17 mutant demonstrated that binding of all three antibodies was recovered (Fig. 3A). To narrow down the number of residues required for antibody binding, we further decreased the number of human amino acids from ECL4 and 6 to nine and generated an HHMM-ECL4&6-h9 chimera (Fig. 3B). In this mutant, five and four mouse amino acids are replaced with the human counterparts in ECL4 and ECL6, respectively. Staining HeLa cells expressing this chimera with UIC2, MRK-16 and 4E3 antibodies showed reactivity to all antibodies.

We next explored if residues from either ECLs 4 and 6 are essential for binding of antibodies. For this, we generated two new chimeras in which residues in ECL4 or ECL6 were replaced. HHMM-ECL6-h4 (Fig. 3C), and HHMM-ECL4-h5 (Fig. 3D) proteins were expressed in HeLa cells and the reactivity of the three antibodies was tested. Interestingly, while HHMM-ECL6-h4 was unable to bind to any of the three antibodies, replacing only five amino acids in the ECL4 of the HHMM chimera efficiently recovered the binding of all three antibodies. These findings demonstrated that substitution of only five mouse amino acids with corresponding human residues in ECL 4 of the HHMM chimera is sufficient to fully recover binding of the three antibodies.

We next determined the ECL residues in the N-terminal half required for recognition by the three antibodies. While all the residues in ECL2 and many of the residues in ECL3 are conserved, ECL1 in human P-gp has a significant number of amino acids that are different from the mouse transporter. In addition, ECL1 of human P-gp has three additional amino acids (M89, S90 and N91) that are missing from the ECL1 of mouse protein. Also, although not playing any role in binding of the three antibodies, there are three N-glycosylation sites (N91, N94, and N99) in ECL1^23^. Considering previous reports on the role of ECL1 in forming UIC2 and MRK-16 epitopes, accompanied by the fact that this loop is the longest and most dissimilar between mouse and human P-gp (Fig. 1), we next proceeded to identify the ECL1 residues forming the epitope recognized by the three antibodies.

Based on alignment of human and mouse P-gp sequences, the three extra amino acids (M89, S90, and N91) of human P-gp ECL1 were added to the MMHH chimera and S86 and M94 of mouse ECL1 were replaced with human E86 and R95 residues to generate the MMHH-ECL1–5h mutant (Fig. 3E). Treating HeLa cells expressing this mutant with antibodies revealed that five amino acid substitutions in the ECL1 was not sufficient to recover a detectable level of UIC2 binding (Fig. 3E, second column). On the other hand, both MRK-16 and 4E3 antibodies were able to bind to this chimera (Fig. 3E third and fourth column). The reactivity of both MRK-16 and 4E3 was about 60–70% compared to WT human P-gp, suggesting that additional residues are required for complete recovery of the epitope of these antibodies. These data indicate an overlapping or common epitope for both MRK-16 and 4E3 antibodies.

### Substitution of thirteen residues in ECL1 and five residues in ECL4 with human P-gp amino acids fully recovered UIC2, MRK-16 and 4E3 antibody binding to mouse P-gp

As noted above, the ECLs of both halves of P-gp are required for the binding of the three antibodies, UIC2, MRK-16, and 4E3. Utilizing mutagenesis and chimeras of human-mouse P-gp, we identified five amino acids in ECL4 of human P-gp that are essential for binding to these antibodies. Next, we wanted to identify residues in ECL1 from human P-gp that could fully recover the binding of all three antibodies to mouse P-gp. For this purpose, we substituted thirteen amino acids in ECL1 of mouse P-gp with human residues, in addition to the five amino acids from ECL4 (Fig. 3D), generating a mouse P-gp with a total of eighteen human residues, and named this chimera mouse P-gp-ECL1&4-h18 (Fig. 4A). HeLa cells expressing mouse P-gp-ECL1&4-h18 demonstrated recognition of this mutant transporter by UIC2, MRK-16 and 4E3 antibodies comparable to the same level as human WT P-gp (Fig. 4B). The total cell expression level of some of these chimeras detected by immunoblotting with C219 monoclonal antibody is given in Supplementary Fig. 2. These results indicate that either the lack of glycosylation as previously reported^23^, or its alteration has no effect on recognition of P-gp by these three antibodies.

Although we were able to fully recover binding of all three human P-gp-specific antibodies to the mouse-ECL1&4-h18 mutant, *in silico* docking analysis suggested the presence of an extra amino acid in ECL3 (G324) that is located in close proximity to the UIC2-Fab (within less than 3 Å distance) that may be involved in binding^21^. Additionally, Zhou *et al*. reported that the deletion of the third extracellular loop between TMH5 and TMH6 abolishes UIC2 binding^29^. Thus, we generated a new mouse P-gp mutant that had an extra amino acid replaced, K323(h324)G, in addition to the eighteen residues we discussed above, and named it mouse P-gp-ECL134-h19 (Fig. 5A). As expected, the binding of all three antibodies to this mutant protein in HeLa cells was fully recovered. For MRK-16 and 4E3, we observed a similar level of binding; however, UIC2 antibody showed higher reactivity to this mutant transporter compared to both human WT and mouse P-gp-ECL1&4-h18 (Fig. 5B). The list of substitutions in all chimeras along with the level of binding of antibodies is given in Table 1.

### Characterization of UIC2 and MRK-16 binding to mouse P-gp with 18 or 19 human residues in ECL 1 and 4

As described above, by substitution of eighteen (ECL 1&4-h18) or nineteen (ECL 13&4-h19) mouse P-gp residues with human P-gp amino acids, we recovered the binding of three antibodies to the same level as human WT P-gp. Since UIC2 antibody showed a higher reactivity to mouse P-gp-ECL134-h19 than mouse P-gp-ECL1&4-h18, we measured the apparent binding affinity of both UIC2 and MRK-16 antibodies to these mutants (Supplementary Fig. 3). Comparing the apparent dissociation constant (K_d_), MRK-16 binding affinities for both mouse P-gp mutants are comparable to those of human P-gp (apparent K_d_ = 9.9 ± 3.6 nM). However, UIC2 binds with slightly higher affinity to mouse P-gp-ECL134-h19 (K_d_ = 5.1 ± 1.9 nM) compared to the human WT (K_d_ = 9.5 ± 1.3 nM) or mouse P-gp-ECL1&4-h18 mutant (K_d_ = 11.7 ± 2.2 nM). We also measured the affinity of MRK-16 antibody for the MMHH-ECL1-h5 chimera (Supplementary Fig. 4).

During drug transport, P-gp undergoes conformational changes that promote drug movement along the translocation pathway and facilitate the efflux of substrates from the cells. There are at least two major states of P-gp, inside-open (inverted V shape) with NBDs separated from each other, and inside-closed (V shape) with dimerization of NBDs and a change in conformation of transmembrane helices and ECLs^30^. Unlike MRK-16 and 4E3, the UIC2 antibody has different reactivity to P-gp in different conformations^25^ and its interaction with P-gp is modulated by pretreatment with substrates and modulators^17^. This reactivity change is measured by the “UIC2 shift assay”^25^. The pre-treatment of P-gp with substrates, such as cyclosporine A at 20 µM, increases the UIC2 reactivity. To test whether the substrate-dependent conformation sensitivity of UIC2 binding is recovered for the mouse P-gp-ECL1&4-h18 and mouse P-gp-ECL134-h19 mutants, we performed the UIC2 shift assay as described in the Materials and Methods section (Fig. 6A–C). Pre-treatment of HeLa cells expressing human WT P-gp or mouse P-gp-ECL1&4-h18 chimera with cyclosporine A increases UIC2 binding by 2- to 2.2-fold and by 1.4- to 1.7-fold, respectively. However, cyclosporine A pre-treatment of mouse P-gp-hECL134-h19-expressing HeLa cells showed only a marginal (1.2- to 1.4-fold) increase in UIC2 antibody binding, which could be because of higher affinity of antibody for this chimera (Supplementary Fig. 3). Nevertheless, it is clear that the conformation sensitivity of UIC2 was also recovered in mouse P-gp by substituting 18 or 19 residues with human P-gp amino acids in ECL 1, 3 and 4.

### *In silico* docking of Fab of MRK-16 and UIC2 to the homology models of MMHH-ECL1-h5 and mouse P-gp-ECL1&4-h18 chimeras

**Figure 7:**
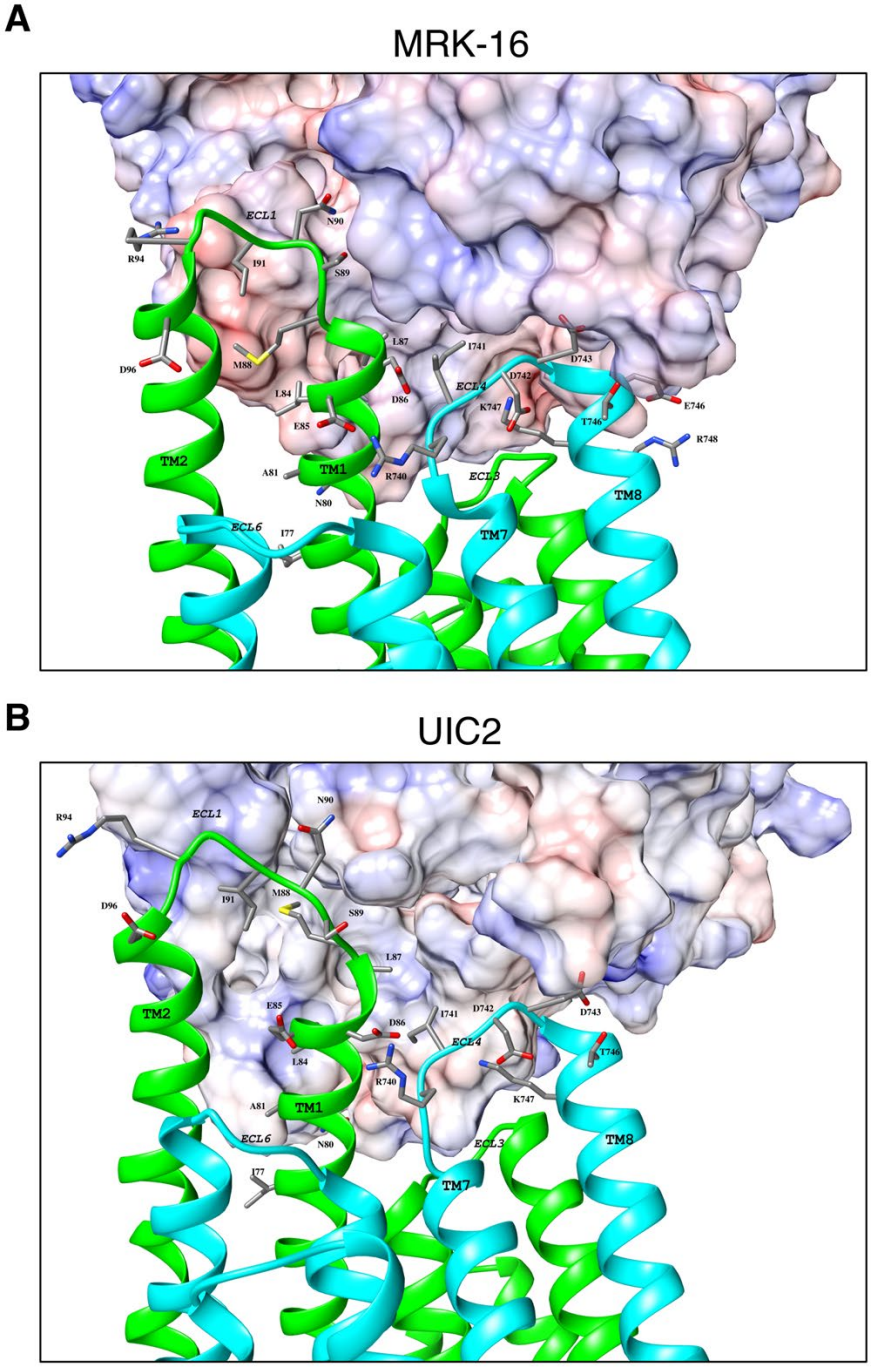
Docking of UIC2 and MRK16 Fab to mouse P-gp-ECL1&4-h18 chimera. The Fab of UIC2 and MRK-16 were docked separately on the homology model of mouse-ECL1&4-h18 P-gp mutant. The extracellular portions of P-gp, which interact with the MRK-16 (**A**) or UIC2 (**B**) Fabs are magnified. The Fab regions are shown as electrostatic surface potentials. The N-terminal half of the P-gp molecule is in green and the C-terminal is in cyan. The ECLs, transmembrane regions, and interacting residues are labeled. The substituted human residues interacting with Fab regions are shown as sticks. These figures were prepared using Chimera version 7.

To gain insight into the interaction of ECL residues of P-gp chimeras with Fab heavy and light chain residues, we performed docking of the Fab structure of MRK-16 and UIC2 antibodies to the homology models of mouse-ECL1&4-h18 P-gp (Fig. 7) or MMHH-ECL1-h5 (Supplementary Fig. 5). We found that the distance between ECL4 and the heavy chain increased for the MMHH-ECL1-h5 chimera, hence there was less contact between the two molecules (Supplementary Fig. 5). This might explain the reduction in the binding of MRK-16 and 4E3 to this chimeric P-gp. Our docking results further showed the importance of the substituted human P-gp residues for interaction with these antibodies (We could not dock 4E3, as the sequence and structure of the Fab of this antibody is not yet known).

For example, our docking analyses showed that R94 in mouse P-gp-ECL1&4-h18 and MMHH-ECL1-h5 can form a salt bridge with residue D61 in the heavy chain of MRK-16. The same residue in model mouse P-gp-ECL1&4-h18 is within a hydrogen bonding distance with Y60 in the heavy chain of UIC2. Similarly, the D743 residue in ECL4 can form a salt bridge with K50 in the light chain of MRK16 (in both P-gp mutants). The same residue (D743) can also form a salt bridge and a hydrogen bond with K55 and Y37 of UIC2’s light chain respectively, but only in the mouse P-gp-ECL1&4-h18 model. In the MMHH-ECL1-h5 model, this residue is within a hydrogen bonding distance with N33 of the light chain.

Our docking analyses further revealed a slightly different antibody binding orientation of MRK16 to mouse-ECL1&4-h18 mutant P-gp compared to the MMHH-ECL1-h5 chimera. In the antibody orientation observed in MMHH-ECL1-h5 chimera docking, the heavy chain of the MRK-16 Fab does not swing towards the transmembrane region, which consequently increases the distance between the light chain and ECL4. This results in loss of binding network in ECL4, which is compensated for by additional new hydrogen bonds such as N90 (h91) and L87 (h88) in ECL1 with W100 and Y95 of the MRK16 heavy chain, respectively. These differences might explain the lower binding affinity of MRK16 observed for MMHH-ECL1-h5 when compared to human WT P-gp. However, it is important to note that due to the flexible nature of the ECL 1 and 4 loops, this speculation will need to be validated by resolving the atomic level structure of the complex of MMHH-ECL1-h5 and the Fab of MRK-16.

We observed that MRK-16 possesses two independent and complimentary features, missing in UIC2, which may explain this difference in binding. The first is P60.H and P95.L, which form a semi-rigid pocket that allows M88, S89 and N59 to fill the volume between the chains. The second is three successive glycines in MRK-16′s heavy chain (G53, G54 and G55) that allow the heavy chain enough flexibility to interact with the P-gp residues closer to the membrane region, including formation of a hydrogen bond between P-gp N80 and N56 of the MRK-16 heavy chain.

A closer look at docking poses showed that there are a few P-gp residues that do not directly interact with Fab, but nonetheless might be essential for antibody interactions. The salt-bridges between ECL4-R740 (h741) and ECL1-E85 (h86) in the mouse P-gp-ECL1&4-h18 chimera/MRK-16 (Fig. 7A) complex, and with ECL1-D86 (h87) in the complex with UIC2 (Fig. 7B) are examples. These interactions most likely keep both extracellular loops 1 and 4 in close proximity to form the epitope and further facilitate antibody binding. It is interesting to note that in the mouse P-gp, D86 of human P-gp is replaced with a lysine residue, which may repulse the R740 side chain. This *in-silico* docking analysis further supports the importance of the mutated residues in mouse P-gp to recover the recognition by the three antibodies.

## Discussion

Chemo-resistance is a major obstacle in cancer treatment. P-gp plays an important role in the development of multidrug resistance in cancer cells by facilitating the intracellular efflux of chemotherapy drugs^6^. For many years researchers explored ways to inhibit cellular processes that promote P-gp expression and/or function, to re-sensitize cancer cells to chemotherapy. Three human P-gp-specific monoclonal antibodies have been developed that target the extracellular regions of the transporter. Although studies have shown that P-gp function can be inhibited by these antibodies, little is known about the nature of the binding or the residues of the P-gp extracellular loops that form the epitopes. In this study, we utilized a human-mouse P-gp chimera approach to map the epitopes of the three human P-gp-specific antibodies UIC2, MRK-16 and 4E3. All human-mouse chimeras studied here (Table 1, Figs 2–5) are expressed at the cell surface of HeLa cells, similar to WT human and mouse P-gp. In addition, the human-mouse P-gp chimeras tested in this work transport rhodamine 123 with the same efficiency as WT proteins (Supplementary Fig. 1), demonstrating that the overall expression, conformation and function is not altered to any detectable level by a major exchange of domains or substitution of residues in the ECLs of human and mouse P-gp, consistent with earlier studies^31^, including those involving major domain interchange of human MDR1 (ABCB1) + MDR2 (ABCB4)^32,33^.

For the first time, we have identified five key residues each in ECL1 and ECL4 (a total of ten residues) of human P-gp that are necessary for the recognition by both MRK-16 and 4E3 antibodies (Fig. 3E). However, these ten residues alone were not sufficient to recover UIC2 binding to mouse P-gp. Extending the number of human residues replaced in mouse P-gp from five to thirteen in ECL1 (in addition to five residues in ECL4), we were able to fully recover binding of UIC2 and MRK-16 and 4E3 as well (Figs 4–5). Thus, our findings indicate that the UIC2, MRK-16 and 4E3 antibodies bind to a discontinuous epitope requiring residues from ECL1, ECL4 and possibly also ECL3. Molecular modeling analyses provided further support for the importance of the mutated residues in ECL1 and ECL4 of mouse P-gp for the interactions of antibodies with P-gp.

Analyzing the concentration-dependent binding profile (Supplementary Fig. 3), we found similar apparent binding affinity of MRK16 and UIC2 antibodies for human WT and mouse P-gp-ECL1&4-h18 mutant P-gp. Interestingly, although there is no change in the affinity for MRK-16, the UIC2 antibody exhibits somewhat higher affinity for the mouse P-gp-ECL134-h19 mutant compared to WT human P-gp (h19 K_d_ = 5.1 vs. WT K_d_ = 9.5 nM; Supplementary Fig. 3). The apparent affinity of the MRK-16 binding to the MMHH-ECL1-h5 chimera was also similar to that of human P-gp (Supplementary Fig. 4).

Using the “UIC2 shift assay”^28^, we observed that the conformation-sensitive binding of UIC2 was recovered when HeLa cells expressing the mouse P-gp-ECL1&4-h18 or mouse P-gp-ECL13&4-h19 mutants were pretreated with cyclosporine A (Fig. 6). These results on modulation of conformation of mouse P-gp containing only 18 or 19 human P-gp residues demonstrate complete recovery of the discontinuous epitope of UIC2 in these mutants.

Although we identified key residues in ECL 1, 3 and 4 interacting with UIC2, MRK-16 and 4E3 antibodies, we cannot rule out the possibility of the involvement of other residues that are common to both human and mouse P-gp. The conserved amino acids between human and mouse could also be important for optimal binding of these antibodies.

In a recent UIC2-Fab-bound cryo-EM structure of mouse-human P-gp chimera in which all extracellular loops were human residues, Alam *et al*.^21^ identified twenty-three amino acids in the ECLs 1, 3 and 4 that are located within 4 Å distance of UIC2 Fab. While six of these amino acids are conserved in both human and mouse P-gp, the rest are different. Comparing these findings with our results, we identified 13 amino acids in ECL1, eight of which are also detected in the cryo-EM structure^21^. Additionally, both we and Alam *et al*. identified five amino acids in ECL4. Consistent with the structural work with a mouse-human P-gp chimera, which found five amino acids in ECL3 that are important for UIC2 binding, our docking analysis also suggests the importance of G324 for binding to all three antibodies. After a closer look at this loop, we observed that 7 out of 10 residues are conserved in both human and mouse P-gp (Fig. 1B). Therefore, it is possible that neighboring conserved amino acids compensate for the slight changes in this loop and UIC2 binding recovery in the mouse-human P-gp chimera (mouse P-gp-ECL1&4h18).

In a previous attempt to identify the MRK-16 binding epitope, Georges *et al*.^22^ compared the reactivity of MRK-16 to P-gp (ABCB1) and ABCB4 (MDR2), which exhibit high amino acid sequence similarity (Supplementary Fig. 6). Utilizing an ELISA-based technique with a series of peptides with overlapping sequences, Georges *et al*. reported that the MRK-16 binding epitope required 11 residues (N106-Y116) from ECL1, and 10 amino acids (T740-R749) from ECL4. We found that the N106-Y116 of ECL1 region does not contribute to the epitope of MRK-16. In ECL4 however, the five key amino acids that we identified to be crucial for binding of MRK-16, UIC2 and 4E3 are among those reported earlier^22^.

Taken together, our results identified the discontinuous epitopes for three monoclonal antibodies that recognize human P-gp. It is noteworthy that the epitope for both the MRK-16 and 4E3 antibodies is located in the same region of ECL1 and 4, whereas the epitope for UIC2 is more complex, including five residues from ECL4, an additional eight residues from ECL1 and also possibly a few from ECL3. It is important to test whether it will be possible to generate antibodies with a continuous epitope involving only either ECL1 or ECL4 by using P-gp fixed in the inside-closed (V shape; ADP-vanadate trapped) conformation as an antigen, where these ECLs would not be in close proximity to each other. For the use of these antibodies in the clinic to inhibit the function of P-gp, the development of single chain antibodies of UIC2 or MRK-16 could be helpful to increase their inhibitory potency.

## Materials and Methods

### Chemicals

Sodium butyrate and other reagents, unless specified, were purchased from Sigma-Aldrich (St. Louis, MO). Cyclosporine A was obtained from the Alexis Corporation (Lausen, Switzerland). Rhodamine 123 was purchased from ThermoFisher (Carlsbad, CA). 4E3 antibody was purchased from Abcam (Cambridge, MA). MRK-16 antibody was obtained from Kyowa Medex Company (Tokyo, Japan). UIC2 antibody was prepared from hybridoma cells as described in the text. FITC-labeled anti-mouse secondary antibody IgG2a was obtained from BD Biosciences (San Jose, CA).

### Cell lines and culture conditions

HeLa cells were purchased from American Type Culture Collection (ATCC, Manassas, VA, USA), and were maintained in Dulbecco’s modified Eagle’s Medium (DMEM) supplemented with 10% Fetal Bovine Serum (FBS), 5 mM L-glutamine, 50 units/ml penicillin, and 50 µg/ml streptomycin at 37 °C^34^.

### Mutagenesis

Several strategies were used to identify amino acids in the extracellular loops to determine the epitopes for UIC2, MRK16 or 4E3. The human-mouse P-gp chimeras (the list with substitutions is given in Table 1) were generated by using gene synthesis (GeneArt services, Life Technology, Carlsbad, CA) as described previously^31^.

### Recombinant BacMam baculovirus generation

The Bac-to-Bac Baculovirus Expression System (Life Technologies, Carlsbad, CA) was used to generate recombinant BacMam baculovirus as described previously^27,31^.

### Expression of human-mouse P-gp chimeras in HeLa cells using the BacMam baculovirus system

HeLa cells were transduced with the human or mouse WT and human-mouse P-gp chimera BacMam baculovirus at a titer of 20–150 particles per cell as described previously^27,31^. Twenty-four hours post-transduction, HeLa cells were harvested and analyzed for cell surface expression and rhodamine 123 transport function. Untransduced and indicated P-gp variant expressing cells (300,000/tube) were analyzed for cell surface expression by incubation for 30–60 min at 37 °C with control mouse IgG2a or human P-gp-specific MRK16 (1 µg/100,000 cells), UIC2 (2 µg/100,000 cells), or 4E3 (0.5 µg/100,000 cells) antibody. Cells were then washed with IMDM medium containing 5% FBS and incubated for another 30 min at 37 °C with FITC-labeled anti-mouse secondary antibody IgG2a (1 µg per 100,000 cells). For the UIC2 shift assay described in Fig. 6, cells were pretreated with 20 µM cyclosporine A before addition of UIC2 antibody. The stained cells were analyzed by flow cytometry using a FACS CANTO II instrument, and the data were analyzed using FlowJo software (Tree Star, Inc. Ashland, OR).

### Transport assay

The transport function of different P-gp chimeras was determined using flow cytometry, as previously described^35^. Cells were incubated with rhodamine 123 at 1.3 µM concentration for 45 min at 37 °C. Cells were washed with cold IMDM supplemented with 5% FBS and re-suspended in cold PBS containing 1% BSA. The transport of rhodamine 123 was measured with respect to the untransduced HeLa cells with an undetectable level of endogenous P-gp, as described earlier^35^.

### Measuring antibody binding affinity

HeLa cells expressing human P-gp, mouse P-gp-hECL1&4-h18 or mouse P-gp-hECL134-h19 (300,000 cells/assay) were stained with different concentrations of each antibody for 30 min at 37 °C. For MRK16 antibody was tested at 0.05, 0.1, 0.25, 0.5, 1, and 1.5 µg per 100,000 cells. For UIC2, we tested 0.01, 0,05, 0.1, 0.25, 0.5, 1, 2, and 3 µg antibody per 100,000 cells. Data analysis and dissociation constant calculations were performed using GraphPad Prism Version 8.

### Homology model of human WT and human-mouse P-gp chimeras and docking of Fab of MRK16 and UIC2 antibodies

The model mouse P-gp-hECL1&4-h18 was based on the recently published structure of the Apo form of UIC2 Fab complex of human-mouse chimeric ABCB1 (pdb.6FN4)^21^. For the MMHH-ECL1-h5 chimera, the mouse P-gp structure (pdb.5KPI)^36^ was used to generate the TMH1–6 model, while the human P-gp homology model^37^ based on pdb.4M1M^38^ was used to model TMH 7–12. The original mouse ECL1 was removed and instead ECL1 was taken from the cryo-EM structure (pdb.6FN4)^21^ and spliced between TM1 and TM2. The five human residues E86, M88, S89, N90, and R94 were retained, while the rest of the residues reverted back to the original mouse sequence.

For docking of Fab of MRK16^39^ and UIC2^40^, each model was used as a docking platform. The Fab regions of MRK16 and UIC2 were docked in the same orientation as the docking orientation of UIC2 to mouse-human chimera structure (pdb.6FN4). This created the initial input for the exhaustive computer-aided docking program. There were four configurations: mouse P-gp-ECL1&4-h18 + MRK16, mouse P-gp-ECL1&4-h18 + UIC2, MMHH-ECL1-h5 + MRK16, and MMHH-ECL1-h5 + UIC2. For each configuration, we applied Rosetta Snugdock protocol^41^ and generated 1,000 possible docking orientations per configuration. From each run, the orientations with the best interface score were chosen and from this list the best orientation was defined as the orientation that maximizes the number of introduced human P-gp residues that interact with the respective antibody. Due to inherent algorithm limitations, after further inspection minor manual adjustments that did not change the secondary or tertiary structures were performed.

## Electronic supplementary material

Supplementary Figures 1–6
