# Supplementary material for: Mapping discontinuous epitopes for MRK-16, UIC2 and 4E3 antibodies to extracellular loops 1 and 4 of human P-glycoprotein
Source: Sci Rep. 2018 Aug 24;8:12716. doi: 10.1038/s41598-018-30984-8 (PMC6109178; doi:10.1038/s41598-018-30984-8)
Supplement: Supplementary file 1 — Supplementary Figures 1–6 [file 41598_2018_30984_MOESM1_ESM.pdf]

Supplementary Figures

Mapping discontinuous epitopes for MRK-16, UIC2 and 4E3 antibodies to extracellular loops 1 and 4 of human P-glycoprotein

Shahrooz Vahedi, Sabrina Lusvarghi, Kristen Pluchino, Yinon Shafrir, Stewart R. Durell, Michael M. Gottesman, and Suresh V. Ambudkar

Laboratory of Cell Biology, Center for Cancer Research, National Cancer Institute, National Institutes of Health, Bethesda, Maryland 20892-4256, USA

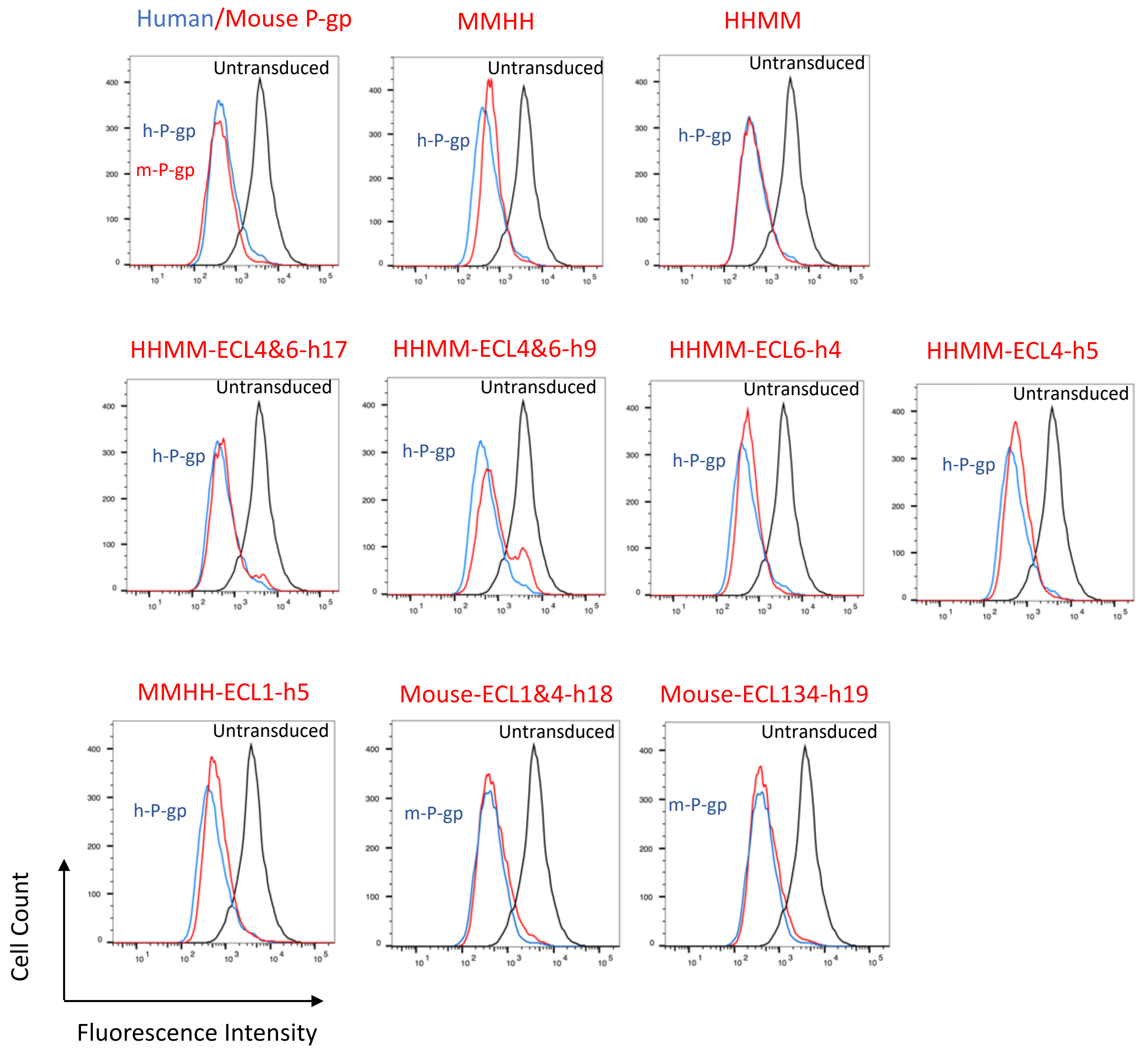

Supplementary Figure 1. Human-mouse P-gp chimeras are functional. The functionality of all human-mouse P-gp chimeras generated in this study was compared by measuring the transport of rhodamine 123. 300,000 untransduced or P-gp variant-expressing HeLa cells were incubated with rhodamine 123 (1.3 μM) for 45 min at 37°C, and the cells were washed and resuspended in PBS containing 1% BSA. The fluorescence intensity was analyzed by flow cytometry using a FACS CANTO II instrument, and the data were analyzed using FlowJo software. In each histogram, black traces with the highest fluorescence intensity represent untransduced cells. Blue traces depict human WT P-gp and mouse WT P-gp (red trace), which were used as controls. The red traces are labeled in each histogram. There is no difference between mouse and human P-gp efficiency in transporting rhodamine 123. The names of chimeras are given above histograms. Representative histograms from a typical experiment are shown. Similar results were obtained in three independent experiments.

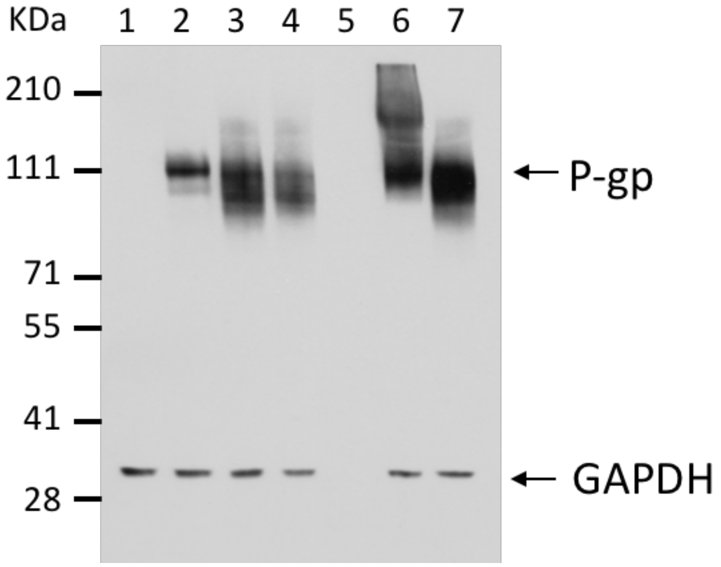

Supplementary Figure 2. Total cell expression level of selected human-mouse P-gp chimeras. SDS-PAGE using 7% Tris-acetate gels was performed with cell lysates of untransduced HeLa cells (lane 1, control), and cells transduced with human P-gp (lane 2), mP-gp-ECL13&4-h19 (lane 3), mP-gp-ECL1&4-h18 (lane 4), mouse P-gp (lane 6), MMHH-ECL1-h5 (lane 7). The lysate of 60,000 cells was loaded in each lane except in lane 5, where the same volume of the 1X sample buffer without cells was loaded. Western blotting was carried out with anti-P-gp C219 at 1: 2000 dilution and anti-GAPDH (6C5 at 1: 20,000 dilution) monoclonal antibodies, followed by anti-mouse HRP-conjugated secondary antibody at 1: 10,000 dilution. SeeBlue-Plus2 pre-stained standard protein markers were used. The blots were developed with ECL reagent (ThermoFisher, Bartlesville, OK) following the manufacturer's protocol. The arrow indicates the position of human P-gp. A Western blot from a representative experiment is shown. Similar results were obtained with two additional independent experiments.

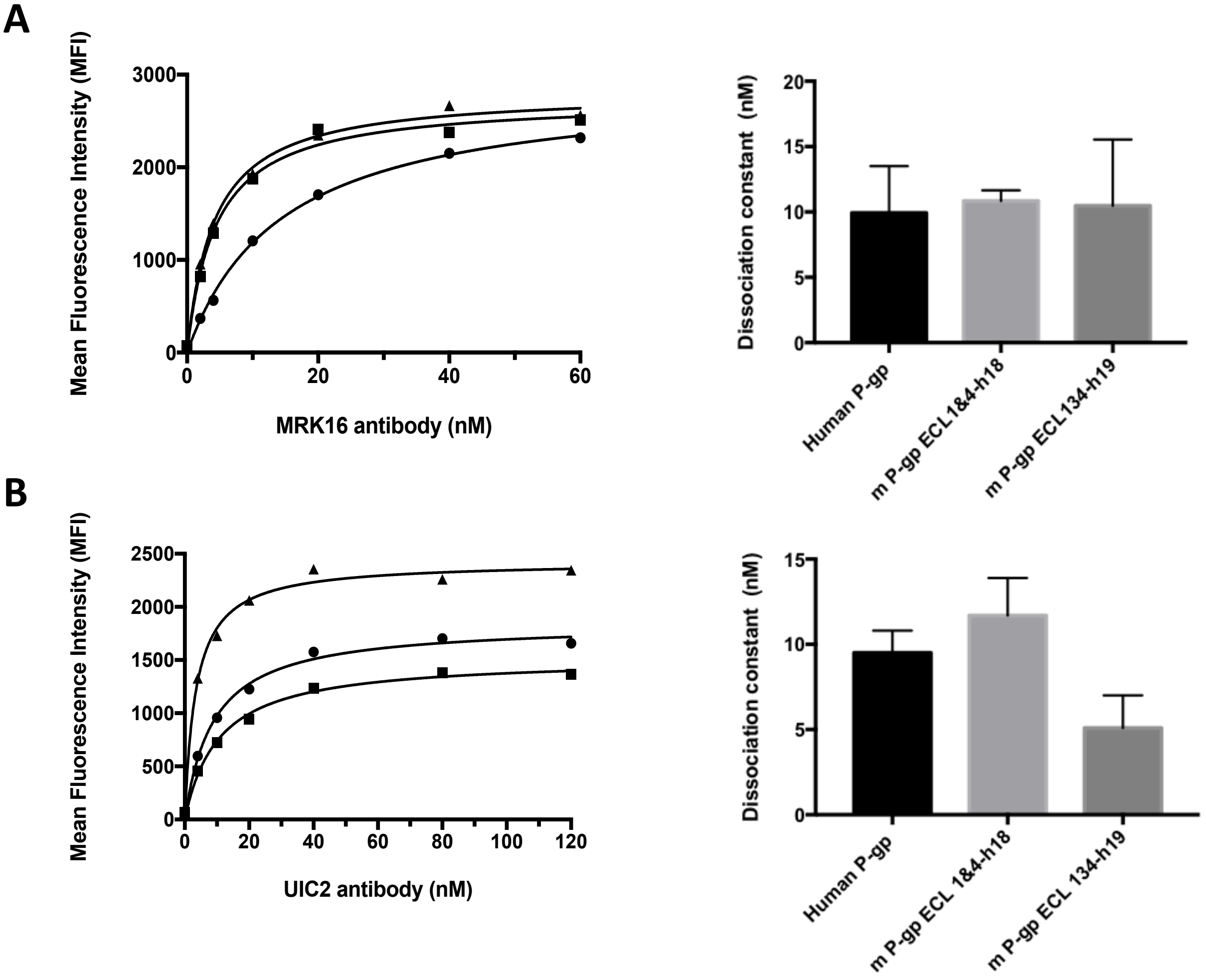

Supplementary Figure 3. The concentration-dependent binding of UIC2 and MRK16 antibodies to mouse P-gp-ECL1&4-h18 and mouse P-gp-ECL134-h19 chimeras. Antibody binding affinity was measured by staining HeLa cells expressing either human WT P-gp, mouse-ECL1&4-h18, or mouse-ECL134-h19 P-gp chimeras at various concentrations of MRK-16 (A) or UIC2 (B). Mean fluorescence intensity was plotted as a function of antibody concentration. Apparent dissociation constant (Kd) values were calculated using GraphPad Prism version 8. Data are from three independent experiments (error bars, SD).

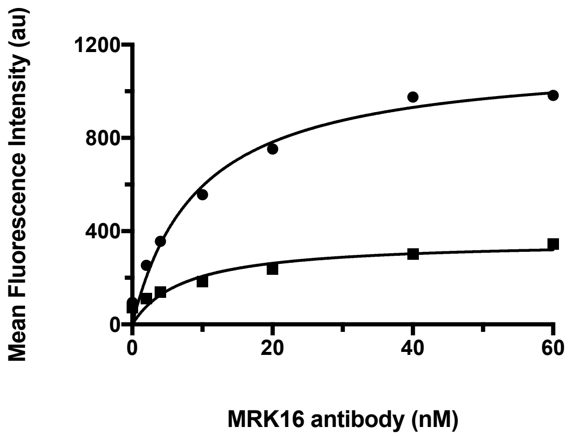

Supplementary Figure 4. MMHH-ECL1-h5 P-gp binds the MRK-16 antibody with a lower affinity compared to human P-gp. MRK-16 binding affinity was measured by incubating human- (circle) and MMHH-ECL1-h5- (square) expressing HeLa cells with increasing concentrations of MRK-16 at 37°C for 1h. Subsequently, cells were treated with FITC-labeled secondary anti-IgG2a antibody and the fluorescence intensity was measured by flow cytometry, as described in Materials and Methods. Apparent dissociation constants were calculated based on two independent experimental replicates using GraphPad Prism Version 8. The values in the graph are the average of the two replicates.

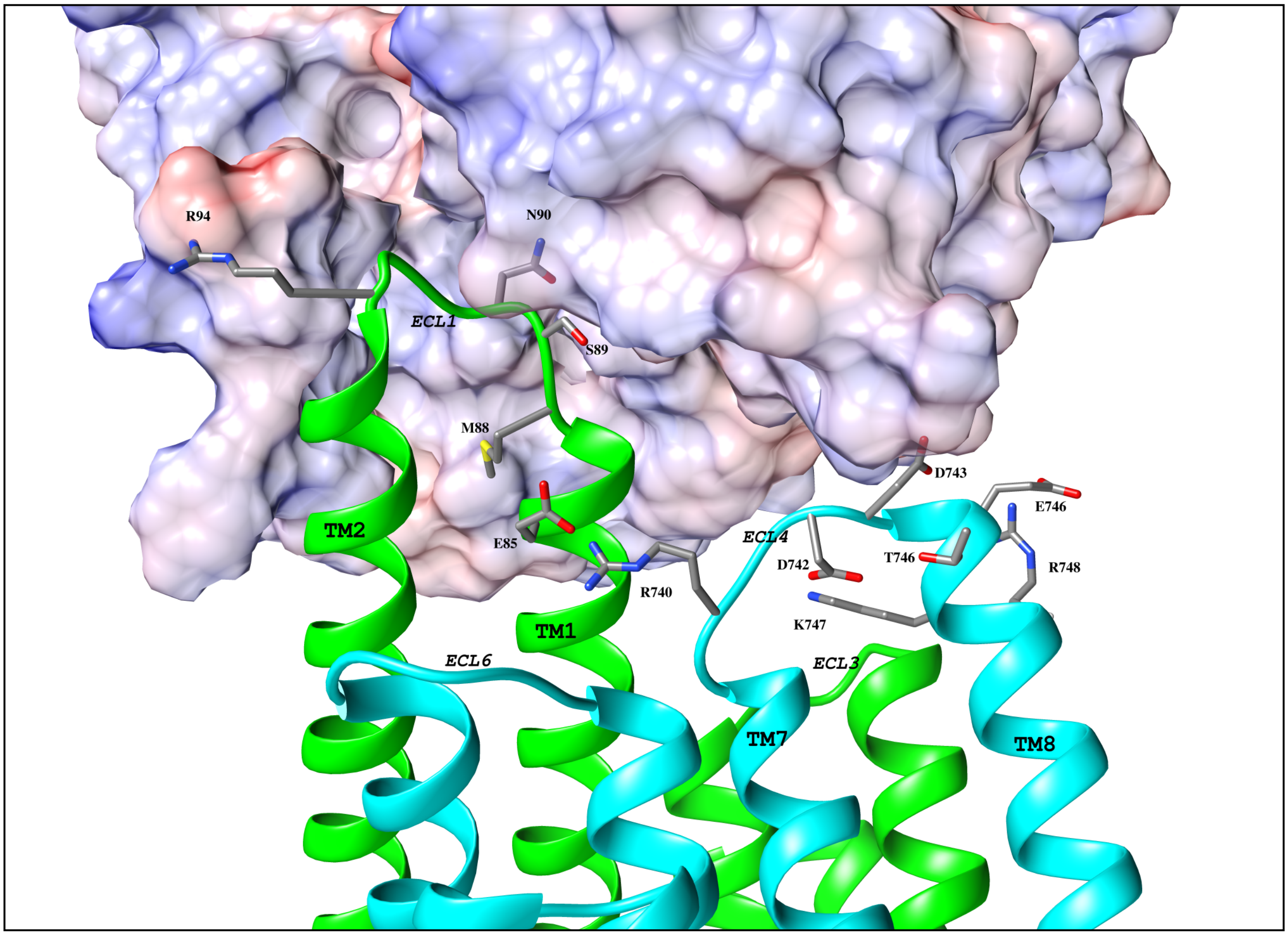

Supplementary Figure 5. The docking of MRK-16 Fab on MMHH-ECL1-h5 chimera. The MRK-16 Fab was docked on the MMHH-ECL1-h5 P-gp mutant as described in Materials and Methods and in the legend to Figure 7. The extracellular portions of P-gp that interact with MRK-16 Fab are expanded. Compared to mouse-ECL1&4-h18, MRK-16 binding is farther from the transmembrane region in this mutant, which decreases the number of protein-protein interactions by about 33%.

ECL1:

73 119

HUMAN_ABCB1 GEMTD**I**FA**NA**GN**LEDLMSNI**TN**R**S**D**INDTGFFMNLEEDMTRYAYYYS

MOUSE_ABCB1A GDMTDSFASVGNVSKN---STNMSEADKRAMFAKLEEEMTTYAYYYT

HUMAN_ABCB4 GEMTDKFVDTAGNFSFPVNFS-L---SLLNPGKILEEEMTRYAYYYS

*:*** *..... .   :   .   ***:** *****:

ECL3:

318 327

HUMAN_ABCB1 TTLVLS**G**EYS

MOUSE_ABCB1A TSLVISKEYS

HUMAN_ABCB4 STLVISKEYT

::**:* **:

ECL4:

732 756

HUMAN_ABCB1 FSKIIGVFT**RIDD**PET**K**RQNSNLFS

MOUSE_ABCB1A FSKVVGVFTNGGPPETQRQNSNLFS

HUMAN_ABCB4 FSEIIAIFGPGDDA-VKQQKCNIFS

:::.:*   .   .::*:.*:

Supplementary Figure 6. Amino acid sequence alignment of the first, third and fourth extracellular loops of human ABCB1 (P-gp), ABCB4 and mouse Abc1a. The sequence alignment of the first, third, and fourth extracellular loops of human P-gp, ABCB4, and mouse P-gp shows conservation of residues. Amino acids in bold are mutated in mouse-ECL1&4-h18 and mouse-ECL134-h19 mutants.
